# Implementation of risk-sharing contracts as perceived by Spanish hospital pharmacists

**DOI:** 10.1186/s13561-019-0242-x

**Published:** 2019-07-17

**Authors:** Reyes Lorente, Fernando Antonanzas, Roberto Rodriguez-Ibeas

**Affiliations:** Departamento de Economiay Empresa, Universidad de La Rioja, Ciguena 60, 26004 Logrono, Spain

**Keywords:** Hospital pharmacists, Pay-for-performance agreements, Personalized medicine, Risk-sharing contracts, Stakeholders

## Abstract

**Background:**

Concerns about financial sustainability of health systems have promoted the adoption of risk-sharing agreements. Nevertheless, few insights have been derived, due to their confidentiality. The purpose of this study is to analyze to what extent these agreements have been implemented in Spain and the importance of several clinical and management variables concerning their use. We also explore whether risk-sharing agreements promote the adoption of personalized medicine. We give a descriptive analysis based on a questionnaire sent to members of the Spanish Society of Hospital Pharmacy, asking about the implementation of risk-sharing contracts in their hospitals.

**Results:**

There were 80 replies. Implementation of risk-sharing agreements was high (90%), being oncology, neurology, dermatology and infectious diseases the main specialties. The most relevant variables were the number of units of medication per year (89%) in price-volume agreements, and the efficacy and uncertainty of treatments (over 75%) in pay-for-performance agreements. Price-volume agreements were suitable for both conventional and personalized medicine and pay-for-performance more specific for personalized medicine. Paying for performance promotes genetic testing (85%).

**Conclusions:**

The results suggest health authorities should encourage the assessment of financial and health outcomes of real-world contracts of conventional and personalized medicine to better know the variables influencing their use.

## Background

Over the last twenty years, public health care systems have adopted several management tools to cope with increasing pressure by pharmaceutical firms to introduce new products, usually at high prices, in a context of uncertainty and budget restrictions. These tools have been variously called money-back guarantee schemes, managed entry agreements, coverage with evidence arrangements, pay-for-performance, outcome-based payments, or price-volume agreements. Throughout this paper, we use the term risk-sharing agreements to refer to both price-volume agreements (mainly intended to ease the financial burden) and pay-for-performance agreements (that link drug payments to clinical outcomes). They are intended to facilitate access to new health technologies when there are uncertainties about clinical results (e.g. medium-run efficacy of treatments and potential target population) and budgetary implications (e.g. duration of treatments). In this sense, major concerns currently come when there are uncertainties about how the efficacy–as shown in clinical trials- can be generalized to real-world medical practice. By implementing these contracts, both the health administration and the pharmaceutical firms share the uncertainty about financial and clinical effects, and benefit from faster access to new products. Depending on the types of these agreements, pharmaceutical firms may either apply discounts related to sales volume (price-volume agreements), or total or partial paybacks when treatments are not as effective and safe as initially believed (pay-for-performance agreements). Thus, pricing and public funding are linked to financial and clinical outcomes [1–6].

Financial sustainability of health systems is a major concern, given the spiraling of health costs caused by new technologies as well as by increasing demand for health services mainly due to an aging population in western societies. Furthermore, as health authorities and pharmaceutical firms are risk-averse, risk-sharing contracts can be understood as means for ensuring the inherent financial and clinical risks of implementing a new treatment are kept to a minimum. As these features will likely be present in the coming decades, it is to be expected that these contracts will become widespread in the future and are likely to be extended to other technologies [7–9].

Risk-sharing contracts are tailored to specific healthcare contexts (hospital, regional, national) as well as to technologies and medical specialties. Nevertheless, there are therapies where the application of these contracts is not straightforward or even necessary. For interventions with low budgetary implications (low prices and small target population), the management costs of these contracts will likely be higher than the savings and so they may not be appropriate. Likewise, if monitoring health outcomes is costly (due to a large target population, for instance), again risk-sharing agreements based on paying for performance may not be appropriate [1, 7].

There is a long tradition of financial agreements between manufacturers and health care systems. However, agreements based on health outcomes have been adopted more recently; the first agreement of this type was adopted in 2007 in the UK for the drug bortezomib. The pharmaceutical firm committed to a payback if the patient did not meet the efficacy rate agreed [10]. In Spain, at the local level, risk-sharing agreements (mainly price-volume) have been used only for hospital-dispensed drugs, and have traditionally been managed by hospital pharmacists, who are responsible for the hospital drug budget. Lately, there have also been new pay-for-performance agreements between hospitals and manufacturers, as well as between regional and national health authorities and pharmaceutical firms. In Spain, until 2011 there were no outcome-based agreements. The first one at hospital level was in Granada, for Volibris (ambrisentan) [11]. Afterwards, other contracts, mostly related to cancer conditions, have been signed between regional health authorities and manufacturers.

Together with the wider use of risk-sharing contracts, the new paradigm of personalized medicine has emerged in the last decade to match treatments to patients’ characteristics with the help of genetic biomarkers. Stratification of patients reduces uncertainties about clinical and financial outcomes of medications. Hence, it could be thought that risk-sharing agreements are not suitable or even necessary in this new context; however, this type of agreement can be used to provide firms with incentives to generate more information on patient characteristics to improve clinical outcomes. From a theoretical perspective, Antonanzas et al. [12] have analyzed the use of risk-sharing contracts to promote personalized medicine. However, from an empirical point of view, the relationship between this type of agreements and the implementation of personalized medicine requires more research, as this topic has not yet been addressed in the literature.

Studies on risk-sharing contracts have focused on defining their types and applicability [3–5, 13–17], on analyzing their characteristics and desirability from a theoretical perspective [6, 7, 12, 18–24], on summarizing real cases and their economic consequences [25–30] and on understanding the perceptions of the stakeholders involved in these contracts [31–34]. However, as Nazareth [34] remarked, despite the frequent use of risk-sharing agreements, little can be learned from them, as the information is not disclosed for confidentiality reasons.

As has been observed, there are few publications assessing real-world agreements that can give some hints about how they were implemented (e.g. duration, monitoring, data protection and patient records), their objectives, financial terms, and health outcomes. In order to improve the knowledge of the specific features of these contracts, a recent study investigated the perceptions of pharmaceutical decision makers from the five largest EU countries (France, Germany, Italy, Spain and the UK) on several key elements of these agreements [32]. Another study by Nazareth [34] assessed the activity related to these contracts as well as the perceptions of stakeholders in the USA and the same five large EU countries. Both studies had a limited participation by Spanish stakeholders (12 in the former and 1 in the latter of the abovementioned articles).

In Spain, the empirical studies dealing with risk-sharing agreements are scarce. We have only found one publication [35] assessing the results of the risk-sharing contract for gefitinib, in Catalonia; other research by Rojas and Antonanzas [36] focused on the perceptions of several stakeholders in Spain about the adequacy of these contracts as a management tool. They carried out in-depth interviews with 14 health care specialists (hospital pharmacists, laboratory managers and oncologists) to understand the legal and practical aspects of different types of agreements as well as the prospects for their future use. In this study, it was found that most of the contracts were signed at the local level between hospitals and pharmaceutical firms. Hospital pharmacists undertook to manage these contracts; given the limitations of the sample size of this empirical work, additional research is needed to understand better their advantages and disadvantages, and to confirm some of the previous findings in the literature.

To learn more about risk-sharing agreements in the Spanish context, the objectives of this study are to analyze the degree of implementation of the risk-sharing agreements and the relevance of several factors that condition their utilization as perceived by hospital pharmacists who are responsible for their design and follow-up. Furthermore, we also explore whether risk-sharing agreements may promote the adoption of personalized medicine and vice versa. The results of this analysis provide clarity about the current situation of the adoption of risk-sharing contracts in Spain, as well as the perspectives for their future development. Likewise, the study highlights the most relevant elements that guide the management of these agreements.

## Material and methods

Participants were members of the Spanish Society of Hospital Pharmacy - Sociedad Española de Farmacia Hospitalaria- (the professional specialists involved in the procurement decisions regarding the adoption of risk-sharing contracts). They were asked about their opinions and personal experience with risk-sharing contracts. The survey was web-based with closed and semi-closed, multiple choice and scale questions. Questions referred to the degree of implementation of different modalities of risk-sharing contracts (price-volume, paying-for efficacy and paying-for efficiency) and their perceptions about several aspects (medical specialties, managerial variables, and regulatory framework) influencing the adoption of these contracts. The questionnaire had 32 questions. The process of designing the questionnaire had several phases, including the revision of preliminary versions by four experts in hospital pharmacy, who suggested changes in several questions to make them more understandable, until the final version was clear enough to be sent out to all the potential responders, (the questionnaire is available upon request).

The invitations to answer the questionnaire were sent by the secretary of the Spanish Society of Hospital Pharmacy to its 3023 members. The participants sent the responses anonymously although they indicated the region where they practice. The survey was open from December 2017 to March 2018. Responses were analyzed using IMB-SPSS and Microsoft Excel. Only a descriptive analysis was performed due to the limited number of responses finally received.

## Results

In total, 80 participants answered the questionnaire. The sample included all Spanish regions, except the Balearic Islands. The number of responses in each region mirrors somewhat the number of hospitals (i.e. large regions with several hospitals generated more responses than small regions); hence, the sample representativeness was adequate. Almost 90% of respondents worked in a hospital pharmacy and about 12% were employed directly at the administrative services of the regional health authority.

It is remarkable that the level of use of risk-sharing agreements was high, as 90% of responders stated that they currently had some kind of contract of this type with a pharmaceutical firm, and that they would like to renew them or to sign new ones (96%). In addition, 25% of those who did not have any agreement of this type showed interest in signing one in the near future. The duration of the contracts was about one year (97% of responders); half of them believed that the duration of the agreement was not a requirement for their feasibility. Among those who stated that they did not have a risk-sharing agreement, half argued that they would make the clinical and administrative management of drugs more complex and that they were not efficient instruments.

The questionnaire focused on three types of agreements (price-volume, paying-for-efficacy and pay-for-efficiency) and asked for information on different issues related to them. Most of the responders (80%) stated that price-volume agreements were implemented in their organizations, while 20% had payment-for-efficacy and only one responder said that it had signed a payment-for-efficiency contract (notice that more than one type of agreement could be in place in a given center). Oncology (97%), neurology (75%), dermatology (49%) and infectious diseases (46%) were the main medical specialties using these agreements, whose modality was price-volume in 95% of cases. However, the payment-for-efficacy agreements barely represented 5% of the responses in most of the specialties except oncology (20%).

Health authorities (regional or local –i.e. hospitals-) and pharmaceutical firms (as stated by 95% of the responders) should design agreements jointly; only 5% considered that health authorities should unilaterally design the contracts.

Almost all responders (90%) considered that there is no need to change current legislation or enact new regulations to manage price-volume agreements, although for the other contracts new specific regulation or just modifications were required (about 66% and 20%, respectively, for pay-for-efficacy, and 64% and 25%, respectively, for pay-for-efficiency). Responders, independently of the type of contract, considered that these regulations should be national (59%) or regional (61%).

Regarding health outcomes as a basis for any type of risk-sharing agreement, 60% of the responders pointed out that overall survival was perceived as the most influential parameter for signing a contract, followed by progression-free survival (22% of responders) and quality of life (23% of responders); other effects such as a better patient monitoring, therapeutic response and higher access to drugs were mentioned by 18% of participants.

As could be expected, the efficacy and efficiency of health technologies were not considered relevant criteria for price-volume agreements (9% and 5%, respectively). However, the relevance of these criteria for contracts based on efficacy were 96% and 71%, respectively, these figures being 75% and 96% for contracts based on efficiency.

Table 1shows the importance of some health and financial variables for each type of agreement as perceived by responders classified by the type of agreement signed by their hospital. It summarizes the relative importance given by responders to each variable. Notice that each row adds up to 100%, although the absolute number of answers may be larger than the number of participants in the survey as they were asked about the importance of each variable for all types of agreements. For instance, in the first row, the “treatment efficacy” is considered to have little importance when signing a price-volume agreements by those who already have that type of contract (only 3 responders), although they deem it more relevant for paying-for-efficacy (54 responders) and for paying-for-efficiency (45 responders) contracts.

**Table 1:** Importance of health-related variables for risk-sharing agreements

	The hospital has only price-volume agreements			The hospital has payment-for-efficacy or efficiency (with or without price-volume)		
HEALTH RELATED VARIABLES	Price-volume	Paying-for-efficacy	Payment-for-efficiency	Price-volume	Payment-for-efficacy	Payment-for-efficiency
Treatment efficacy	3 (2,9%)	54 (52,9%)	45 (44,1%)	3 (9,4%)	17 (53,1%)	12 (37,5%)
Treatment efficiency	2 (1,9%)	46 (45,1%)	54 (52,9%)	1 (3,7%)	10 (37%)	16 (59,2%)
Duration of the treatment	54 (48,6%)	30 (27%)	27 (24,3%)	15 (50%)	8 (26,7%)	7 (23,3%)
Uncertainty on treatment effectiveness	17 (15%)	52 (46%)	44 (38,9%)	4 (12,5%)	15 (46,9%)	13 (40,6%)
Adverse events and toxicity	13 (12,7%)	51 (50%)	38 (37,2%)	3 (9,4%)	17 (53,1%)	12 (37,5%)
Incremental safety versus the standard of care	9 (8,9%)	53 (52,5%)	39 (38,6%)	2 (7,1%)	14 (50%)	12 (42,8%)
Size of target population	56 (60,8%)	20 (21,7%)	16 (17,4%)	15 (60%)	6 (24%)	4 (16%)
Number of packages of medication per year	56 (88,9%)	4 (6,3%)	3 (4,8%)	16 (94,1%)	1 (5,9%)	0 (0%)
Unitary cost per dose	56 (59,6%)	18 (19,1%)	20 (21,2%)	13 (50%)	6 (23,1%)	7 (26,9%)
High budget impact of the treatment	55 (74,3%)	9 (12,2%)	10 (13,5%)	13 (50%)	6 (23,1%)	7 (26,9%)
Rare disease	30 (34,9%)	44 (51,2%)	12 (13,9%)	10 (41,7%)	10 (41,7%)	4 (16,7%)
Orphan drug	30 (35,3%)	43 (50,6%)	12 (14,1%)	11 (42,3%)	11 (42,3%)	4 (15,4%)
First in class drug	16 (23,9%)	45 (67,1%)	6 (8,9%)	6 (26,1%)	13 (56,5%)	4 (17,4%)
Previous experience of agreements with the laboratory	23 (31,5%)	44 (60,3%)	6 (8,2%)	13 (40,6%)	13 (40,6%)	6 (18,7%)

Responders whose hospitals had only price-volume agreements considered that the number of units of medication per year (88,9%), the high budget impact of the treatment (74,3%), the size of the target population (60,8%), the unitary cost per dose (59,6%), and the duration of the treatment (48,6%) were the most important variables to be taken into account when signing price-volume agreements. With regard to payment-for-efficacy contracts, the responders stated that being the drug “first in class” (67.1%), the efficacy (52.9%) and efficiency (45.1%) of the treatment, and the previous experience with the laboratory or pharmaceutical firm (60.3%) were the most important variables. Finally, for payment-for-efficiency contracts, the respondents perceived the efficacy of the treatment (44.1%) and the uncertainty on the effectiveness of the treatment (38.9%) were the most important variables. As expected, the relative importance of each variable is very close for pay-for-efficacy and pay-for efficiency agreements.

Responders whose hospitals had payment-for-efficacy or efficiency agreements (independently of whether they also had price-volume agreements signed) considered that the number of packages (94.1%), the size of the target population (60.0%), and the units of medication per year (94.1%) were the most important variables that should be taken into account for signing price-volume agreements. Regarding payment-for-efficacy agreement, responders perceived that being the drug “first in class” (56.5%), the efficacy (53,1%) and the uncertainty (46.9%) of the treatments, the adverse events and toxicity (53.1%), and the relative safety compared to the existing treatment (50.0%) were the most important variables for payment-for-efficacy agreements. Finally, for payment-for-efficiency agreements, the efficiency of the treatment (59.2%), the incremental safety (42.8%) and the adverse events and toxicity (37.5%) were deemed the most important variables.

As it could have been expected, variables more directly related to health had a higher importance for payment-for-efficacy and efficiency agreements than for price-volume ones, while finance related variables were more relevant for price-volume agreements, independently of the type of agreement signed by the hospital where responders worked.

Regarding how the agreements were formalized, 28% of the participants stated that they only used a written document, while 4% said that they were only agreed verbally; a combination of both forms was used by 68% of the responders.

Most of the responders (84%) acknowledged that there were administrative advantages to risk-sharing contracts. Budget control was highlighted as the main reason to sign these contracts, followed by better drug management and easier patients’ access to new drugs. Most pharmacists (92%) considered that risk-sharing contracts generated incentives to find new information when health outcomes of new treatments were uncertain.

Responders perceived that the widespread adoption of risk-sharing agreements would lead to more complex protocols and clinical management of patients (55%). However, they were not sure (64%) whether these contracts would make clinical services in the future more dependent on other services (such as genetic laboratory, records department and hospital pharmacy).

Participants remarked that these contracts also had some drawbacks. Table 2 summarizes the perceptions of the respondents about the level of effort (low, medium or high) required to overcome some drawbacks of these contracts. Responders were classified according to the type of contract their hospitals had signed (price-volume only or other types). Responders belonging to hospitals with only price-volume agreements mentioned that most of the drawbacks (building or adapting information systems to manage the agreements, the administrative and legal bureaucracy to draw up the agreements, the decisions about the cost-effectiveness threshold or sales threshold for price reductions and savings, and the administrative follow-up to guarantee compliance with the agreement) would require a medium or a high effort to overcome them. On the contrary, persuading clinicians to prescribe the drugs included in the agreements and keeping patient registries were thought to require low effort. In the case of responders who had payment-for efficacy or for efficiency, their perceptions were of similar nature although a higher percentage of responders seemed to point out as “high” the effort needed to overcome the drawbacks derived from the agreements.

**Table 2:** Level of effort needed to overcome some of the drawbacks of the risk-sharing agreements

Drawbacks	Type of agreement					
	Price-volume			Other		
	Low	Medium	High	Low	Medium	High
Keeping patient records	32.1%	62.5%	5.4%	29.4%	47.1%	23.5%
Persuading clinicians to prescribe the drugs included in the agreements	46.4%	51.8%	1.8%	35.3%	58.8%	5.9%
Building or adapting information systems to manage the agreements	5.4%	80.4%	14.3%	5.9%	52.9%	41.2%
Administrative and legal bureaucracy involved in drawing up the agreements	3.6%	76.8%	19.6%	29.4%	29.4%	41.2%
Decision about the cost-effectiveness threshold or sales threshold for price reductions and savings	16.1%	60.7%	23.2%	17.6%	47.1%	35.3%
Administrative follow-up to guarantee compliance with the agreement	7.1%	78.6%	14.3%	5.9%	58.8%	35.3%

The questionnaire also explored the relationships between risk-sharing agreements and the promotion of personalized medicine. In that sense, participants (85%) stated that price-volume agreements would be maintained in a similar way no matter what the type of medicine (either conventional drugs or those that require genetic tests to personalize treatments); however, the other types of agreements would be more appropriate in personalized medicine (81%).

Only 12% of responders perceived that price-volume agreements would be an incentive for performing genetic testing, while 85% of participants believed that the other type of agreements would provide be an incentive for those tests. Furthermore, when considering incentives in reverse, it turned out that performing genetic tests was believed to lead (80% of responders) to a greater use of risk-sharing contracts of the paying-for-efficacy or efficiency type than the price-volume ones (17%).

Regarding the managerial effort necessary to deal with these contracts when applied to personalized medicine, responders considered that, for price-volume agreements, they are as high as in conventional medicine; however, for the other types of contracts, this effort would need to be greater.

## Discussion

This study has explored the level of utilization of risk-sharing agreements in Spain and the perceptions of hospital pharmacists about the relevance of several elements for their implementation. The questionnaire was distributed through the Spanish Society of Hospital Pharmacy that has over 3,023 members; a reminder was sent later during the survey period to encourage participation. Nevertheless, the rate of response was limited and only 80 responses were valid for the analysis.

In Spain, hospital pharmacists are the professionals most directly involved with decision-making about the design, management and follow-up of these agreements at the local level (i.e. hospitals), although there are a few experiences where the initiative to implement these contracts is taken by regional health authorities (as in Catalonia and Andalusia). Therefore, we believe that the potential selection bias of the sample is low as the questionnaire was addressed to the appropriate group of health professionals. However, there may be some bias because questionnaires tend likely to be answered by hospital pharmacists with some experience in the survey topic. Initially, it would have been difficult to send the questionnaire only to those who have the expertise and also who finally responded, and then get a rate of response much higher. In this respect, for instance, it would be tempting to assume that almost all hospitals have price-volume agreements, as 90% of responders stated that they have that kind of contract, which may not be true in the real world due to that potential bias. However, that bias would not be applicable to other questions related to the factors influencing these agreements, the need to modify the regulations and the relationships with the personalized medicine, as the aggregated results represent choices within a list of variables, and we believe extrapolation errors would not be so dependent on having such contracts or not. Responders were fairly well distributed across the country and we obtained more responses from more populated regions. Spain has 17 regions that manage the health care with an important degree of administrative power, and the responses came from each region but the Balearic Island. Hence, although the final number of responses was not as high as desired in statistical terms, we believe that the distribution of the responses facilitates a broad and comprehensive perspective of the perceptions about these contracts.

Although there was an initial interest in testing some potential associations between variables, statistical inference was not advisable due to the small sample size. Thus, the analysis has been mainly descriptive and exploratory contributing to the scarce literature on the perceptions of the stakeholders. Research on perceptions is a novel and emerging topic, carried out as an alternative to learn about the details of risk sharing contracts, given the lack of transparency about the management and results of such contracts. Other authors working in this area also performed a descriptive analysis based on a limited number of responses. Nazareth et al. [34] based their analysis on 27 responders distributed in six countries, and found that price-volume agreements were generally confidential. Their duration, the sales thresholds, the drugs covered, the related discounts and their impact on cost savings were not usually reported. In addition, Dunlop et al [32], based on 66 responders for five EU countries, remarked that stakeholders considered that these agreements were useful managerial tools and their number would increase in the future.

Although the number of responses is low, as compared to the total number of potential responders of the Spanish Society of Hospital Pharmacy, this sample size is even larger than the sizes used in the aforementioned articles (although these studies do not explicitly stated the sample size, there are more professionals in each country that could have potentially been interviewed). Moreover, as our research only focuses on the Spanish situation, the number of responses is large enough to provide a preliminary picture of the Spanish situation.

There are a few publications on paying-for-performance agreements providing at least information about the drugs covered. To the best of our knowledge, there is only one publication assessing the impact and effects of an agreement in Spain [35].

Besides analyzing the implementation of risk-sharing contracts, this study has also explored the potential synergies between such contracts and the promotion of personalized medicine. In this respect, responders perceived that contracts based on efficacy and efficiency could be an incentive for using personalized treatments. Price-volume agreements are believed to continue being applicable under this new paradigm and, hence, could be applied as currently, but with no synergistic effects to promote personalized medicine.

Price-volume agreements are the most common type and those based on performance are just emerging and are expected to become more prevalent in the future, probably related to the development of personalized medicine. The administrative complexity linked to these agreements together with the need to modify the legal and regulatory framework are perceived as the main barriers to their more widespread use.

Our findings for Spain seem to be in line with the findings of some studies that have recently remarked that health care payers consider price-volume agreements to be the most common type of contract (77% had experience with these contracts, as also found by Dunlop [32]); less than half had experience with paying-for-performance contracts –in their text “innovative agreements”-; oncology was also the area where these contracts are more frequently applied, as detected in our study; the majority of the responders held a positive view about the potential of this type of agreement to increase certainty, manage budgets and provide additional knowledge on the value of the drugs. The study by Nazareth [34] (that only included one Spanish participant in the sample) also highlighted the increasing trend in the use of these contracts in the EU’s five largest countries, again, pointing out that 60% of all contracts signed in the last 20 years addressed cancer drugs, mainly motivated by their growing cost per patient. Responders also gave as a drawback the lack of a clear guiding framework for designing, implementing and monitoring these contracts. That study also anticipates an important increase in the number of these type of contracts in the next five years (as derived from the Spanish survey). As a future research topic, it will be interesting to extend the research scope to other countries and check if these findings hold in other jurisdictions.

As regards the barriers to implementing the contracts, insufficient data infrastructure for monitoring the results and high administrative burdens were pointed out by most of responders.

So far, the empirical literature on risk-sharing contracts has focused on the relevance of several elements that characterize these contracts, as perceived by different stakeholders, mainly due, we believe, to lack of information due to the confidentiality of the terms of the contracts. However, we consider that a more precise understanding of the related variables and factors affecting these contracts should be directly derived from the assessment of real-world contracts, together with a description of their financial and health outcomes results. So far, this kind of assessment has scarcely been published, although perhaps some health care managers for their own use have done it. The lessons from this contracting experience will be crucial for implementing future contracts, and likely popularize their use, even for personalized medicine. That assessment exercise should be encouraged by health authorities, and a unified registry of the main results would be helpful for future development of this type of agreement.

## Conclusions

The level of implementation of risk-sharing agreements in Spain was high, as 90% of responders stated that they currently had this kind of contract with a pharmaceutical firm, and that they would like to renew them or to sign new ones. Responders emphasized that the most common risk-sharing agreements adopt the form of price-volume. Oncology, neurology, dermatology and infectious diseases were the main medical specialties using these agreements.

For hospitals with only price-volume agreements, the influential variables for establishing such agreements were the number of units of medication per year, the high budget impact of the treatment, the size of the target population, the unitary cost per dose, and the duration of treatment. However, for hospitals that had pay-for-performance agreements the influential variables were the efficacy of treatments, the uncertainty about the effectiveness of treatment, the adverse events and toxicity, and the relative safety compared to the existing treatment, as perceived by about 90% of responders.

Budget control was highlighted as the main reason to sign these contracts, followed by better drug management and easier patients’ access to new drugs. Regarding the disadvantages of these contracts, some elements such as keeping patient records, adapting information systems, bureaucracy in drawing up the agreement, and the administrative follow-up to enforce the terms of the contract, among others, would require additional managerial effort.

Participants stated that price-volume agreements would be maintained in a similar way no matter the type of medicine (either conventional drugs or those that require genetic tests to personalize treatments); however, risk-sharing agreements based on outcomes would be more appropriate in personalized medicine. A low proportion of responders perceived that price-volume agreements would promote genetic testing, while the majority of participants believed that the other type of agreements would provide an incentive for those tests. The study also highlights the mutual influence expected to take place between these contracts and the personalization of treatments.

Health authorities should encourage stakeholders to assess the financial and health outcomes of real-world risk-sharing contracts to understand better the variables influencing their use to help future development of these contracts. To facilitate this task and to learn from the experience, a national registry with data of these agreements should be created. If healthcare systems aim to speed the utilization of these contracts, a more clearly legal framework has to be developed to simplify the terms of the agreements, what indeed will reduce the burden on health personnel. This managerial tool, once its use is extended, is believed to improve patients´ access to new technologies.

## Data Availability

Please contact author for data requests.
